# Dementia severity at death: a register-based cohort study

**DOI:** 10.1186/s12888-018-1930-5

**Published:** 2018-11-01

**Authors:** Jesutofunmi Aworinde, Nomi Werbeloff, Gemma Lewis, Gill Livingston, Andrew Sommerlad

**Affiliations:** 1 Division of Psychiatry, University College London, 6th Floor, Maple House, 149 Tottenham Court Road, London, W1T 7NF UK; 2 Camden and Islington NHS Foundation Trust, London, UK

**Keywords:** Dementia, Prognosis, Epidemiology, Health records, Mortality, Death

## Abstract

**Background:**

One third of older people are estimated to die with dementia, which is a principal cause of death in developed countries. While it is assumed that people die with severe dementia this is not based on evidence.

**Methods:**

Cohort study using a large secondary mental healthcare database in North London, UK. We included people aged over 65 years, diagnosed with dementia between 2008 and 2016, who subsequently died. We estimated dementia severity using mini-mental state examination (MMSE) scores, adjusting for the time between last score and death using the average annual MMSE decline in the cohort (1.5 points/year). We explored the association of sociodemographic and clinical factors, including medication use, with estimated MMSE score at death using linear regression.

**Results:**

In 1400 people dying with dementia, mean estimated MMSE at death was 15.3 (standard deviation 7.0). Of the cohort, 22.2% (95% confidence interval 20.1, 24.5) died with mild dementia; 50.4% (47.8, 53.0) moderate; and 27.4% (25.1, 29.8) with severe dementia.

In fully adjusted models, more severe dementia at death was observed in women, Black, Asian and other ethnic minorities, agitated individuals, and those taking antipsychotic medication.

**Conclusions:**

Only one quarter of people who die with dementia are at the severe stage of the illness. This finding informs clinical and public understanding of dementia prognosis. Provision of end-of-life services should account for this and healthcare professionals should be aware of high rates of mild and moderate dementia at end of life and consider how this affects clinical decision-making.

**Electronic supplementary material:**

The online version of this article (10.1186/s12888-018-1930-5) contains supplementary material, which is available to authorized users.

## Background

Dementia is a principal cause of death in England and Wales [1] and the United States [2], and it is estimated that a third of people over the age of 60 die with the condition [3]. Estimates of survival after disease onset have varied between 3.3 and 11.7 years [4] – and the uncertainty over prognosis creates difficulties for patients and their family carers about planning their life and how to prepare for end of life care [5]. The variability in survival time in dementia is most likely a consequence of other illnesses affecting survival. Some studies report a trend in recent years towards compression of morbidity, where the onset of the first chronic illness occurs at a later age, thus compressing the time living with chronic illnesses [6].

There is increasing emphasis on providing good end of life care in dementia, which tends to focus on the challenges of delivering it to people with severe dementia [7]. The proportion of people who have mild, moderate, and severe dementia at end of life has important implications for clinician- and public-awareness of prognosis, how healthcare services plan and implement treatment, and how clinicians provide evidence-based end of life care. Severe dementia is associated with lack of insight ^a^nd capacity [8] to make decisions about healthcare, requiring others to make decisions on behalf of patients [9]. Someone with mild dementia is more likely to be able to make these decisions, but may require support to do so and to implement them. If many people who have dementia die with a mild form of the condition, this would have implications for palliative settings and may suggest a greater need for support for decision making and management strategies. One study reported that 31/68 (46%) people with dementia being managed at end of life by a general practitioner were described by the clinician as having mild dementia, [10] however there has been, to our knowledge, no study examining the distribution of dementia severity in a large cohort of people with dementia at time of death.

In this study, we therefore aim to investigate the severity of dementia at death, in a secondary mental healthcare service cohort of older people with a diagnosis of dementia and explore the association between demographic and clinical factors, including medication use (antidepressant, sedative and antipsychotic drugs, and those for cognition in dementia), and dementia severity at death.

## Methods

### Study setting and data source

We conducted a retrospective cohort study using routinely collected data from clinical dementia services in Camden and Islington NHS Foundation Trust (CIFT), a large mental health trust providing mental healthcare services including dementia assessment and treatment to a catchment area of 470,000 people in two London boroughs. We obtained our data using the Clinical Record Interactive Search (CRIS) system, a platform designed to facilitate the use of routinely collected electronic health records for research, which has been used to address a number of research hypotheses in mental health and dementia research [11]. CRIS allows the extraction of data from electronic health records’ structured and unstructured (e.g. progress notes, clinic letters) fields using the General Architecture for Text Engineering (GATE) program, which uses natural language processing (NLP) algorithms [12], to identify text relating to diagnosis, treatment or other clinical information. The Camden and Islington CRIS database holds pseudo-anonymised electronic mental health records dating from 1st January 2008 for over 116,000 people who have had contact with CIFT services [13]. Individual patient consent is not required for inclusion in the database.

### Study patients

We retrieved records from eligible patients who had clinical contact with CIFT services during the study window (1st January 2008 to 30th September 2016).

Individuals were included if they:

- were aged 65 years or over at dementia diagnosis, ascertained either using a structured field diagnosis of International Classification of Diseases (ICD) 10 codes F00–03 [14], or an unstructured diagnosis (derived though NLP application)
- had their cognitive function assessed and recorded using the Mini-Mental State Examination (MMSE) [15]
- died before 30th September 2016

We excluded patients who received a diagnosis of Mild Cognitive Impairment (MCI) after dementia diagnosis (*n* = 15); this was judged to be an active clinical decision to replace dementia diagnosis with MCI, as it is not possible to have MCI and dementia simultaneously.

### Study data

#### Outcome

Dementia severity at death was assessed using the last recorded MMSE score before death. The MMSE scale is commonly used by healthcare professionals to assess cognitive impairment and monitor progression of decline in people with dementia. It is scored out of 30, and has been found to have acceptable psychometric properties to rate dementia severity [16]. We extracted all recorded MMSE scores from the unstructured fields of patient records, using the NLP application. Each MMSE score was accompanied by a post processing rule (PPR) code which provided information about the quality of the score recording and extraction [17]. A PPR code of 0 indicates that the MMSE has been optimally recorded and codes from 1 to 12 indicated a range of possible problems, such as that the numerator was higher than the denominator, or that more than one record with different scores were recorded on the same date. For all eligible patients, we used the PPR codes to select the valid MMSE records.

We validated the application for extracting and coding MMSE score. We calculated the positive predictive value (PPV) by randomly selecting MMSE scores and dates (*n* = 100) and determining if these were accurate by manually searching through the source documents in which they had been recorded. To obtain sensitivity, we extracted and read through a random set of documents (*n* = 100) which contained the word ‘MMSE’ to ascertain whether there was mention of MMSE score, then determining if this agreed with the coding performed by the NLP. The PPV for MMSE scores was 98%, though the accuracy of dates was substantially lower (67% overall) due to a technical problem which meant that a large proportion were marked with the date of migration to a new electronic clinical record system; so we manually corrected these dates. The sensitivity of the MMSE application was 94%.

#### Covariates

We obtained information on age at diagnosis, sex, ethnicity (White, Black, Asian, and other), marital status and the last recorded dementia subtype (categorised into 5 groups; Alzheimer’s disease, vascular dementia, dementia with Lewy bodies, other, and unspecified). We estimated socio-economic status using the area-level of socio-economic deprivation by matching lower super output area (LSOA) score to the 2010 Index of Multiple Deprivation (IMD) [18]. We obtained information on clinical presentation using patient’s Health of The Nations Outcome Scales (HoNOS) score within a year of diagnosis. HoNOS is a 12 subscale assessment tool with acceptable psychometric properties [19] which is routinely administered at 6 monthly intervals after initial assessment. We used the domains covering the neuropsychiatric symptoms of clinical interest- agitation, hallucination and depressed mood. Because only scores ≥2 are considered clinically significant, we categorised each domain as binary, with 0–1 categorised as ‘no problem’ and 2–4 as evidence of ‘problem’.

Data on recorded medication use (derived from a GATE text extraction) [20] any time after dementia diagnosis, including drugs for cognition (acetyl-cholinesterase inhibitors and memantine), antipsychotics, antidepressants and sedatives (benzodiazepine or ‘z-drug’ hypnotics), were also extracted using NLP application.

### Statistical analysis

We first described the sociodemographic and clinical characteristics of our sample.

#### Estimating dementia severity at death

We assumed that cognition would decline during the time between the last MMSE assessment and death. We therefore estimated MMSE at death by calculating the mean annual MMSE decline using individuals’ last two MMSE scores and, for each patient, multiplying the duration between their last MMSE and death by the cohort’s annual MMSE decline. We report this, as well as the distribution of dementia severity at death, rating MMSE ≥20 points as mild dementia, < 20 and ≥ 10 as moderate, and < 10 as severe dementia, with 95% confidence intervals for these proportions. As we judged that variable interval between last recorded MMSE and death might reduce the accuracy of our adjustment procedure, we conducted a sensitivity analysis using a subgroup of patients who had been assessed close to death (last recorded MMSE score within one year of their death), with the same adjustment procedure.

#### Exploring predictors of dementia severity at death

We examined associations between estimated MMSE scores (continuous outcome variable) and each covariate (predictor variables), using linear regression. We first conducted univariable analyses of the association between each covariate and severity of dementia at death and then our planned primary mutually-adjusted multivariable analysis of the association between these factors and dementia severity at death. Due to a large amount of missing data, with slight differences in sex, ethnicity and socio-economic status between those with complete data and those with missing data, we also conducted an analysis without the HoNOS variables. We also conducted a sensitivity analysis using the same regression analysis on the subgroup whose last MMSE was within a year of their death. In all multivariable analyses, we adjusted for time between diagnosis and death as we considered that this would be a strong confounding factor.

#### Exploring the influence of medication use after dementia diagnosis on dementia severity at death

We conducted univariate and multivariable analyses for associations between our outcome and use of drugs for cognition, antipsychotics, antidepressants and sedatives after dementia diagnosis, adjusting for all the covariates and each medication individually.

All data analysis was completed using STATA (version 11).

### Ethics approval and access to the data

Ethical approval for the analysis of the CIFT CRIS database was obtained from the National Research Ethics Service Committee East of England—Cambridge Central (14/EE/0177).

## Results

The clinical and demographic characteristics of the cohort are displayed in Table 1. Our final cohort consisted of 1400 people. The median age at diagnosis was 84.6 years, and the mean time between diagnosis and death was 1.9 years (standard deviation (s.d.) = 1.7) and maximum time from diagnosis to death was 11.9 years. For those who died with mild dementia, mean time between diagnosis and death was 1.6 (1.4) years; for moderate dementia 1.9 (1.7) and for severe dementia 2.2 (1.9). The majority of individuals were White, and Black people formed the largest minority group. Of those with a recorded diagnosis, Alzheimer’s disease was the most common subtype, followed by vascular dementia. Mean MMSE closest to time of diagnosis was 18.2 (6.6). Around one quarter of patients had clinically significant agitation at diagnosis and one fifth had depressive symptoms. Just under half were recorded as taking medication for cognition in dementia after their diagnosis and 30% took antidepressants.

**Table 1:** Clinical and demographic characteristics of patients (*n* = 1400)

Characteristic		All people with dementia
		n (%)
Age at diagnosis (*years*)	Median (IQR p25, p75)	84.6 (79.4, 88.8)
	Range	65.1–104.3
	*65–69*	44 (3.1)
	70–74	104 (7.4)
	75–79	234 (16.7)
	80–84	349 (24.9)
	85–89	412 (29.4)
	90+	257 (18.4)
Sex	Female	758 (54.1)
Ethnicity	White	1073 (85.0)
	Black	111 (8.8)
	Asian	41 (3.2)
	Other	38 (3.0)
	*Missing*	137
Marital status	Married	388 (33.2)
	Widowed	412 (35.3)
	Divorced	133 (11.4)
	Single	235 (20.1)
	*Missing*	232
Last recorded dementia type	Alzheimer’s disease	645 (46.1)
	Vascular dementia	258 (21.1)
	Dementia with Lewy bodies	32 (2.3)
	Other	135 (9.6)
	Unspecified	330 (23.6)
IMD	Mean (SD)	31.0 (12.6)
	*Missing*	115
HoNOS at diagnosis – problems with*:	Agitation	149 (25.3)
	Hallucinations	95 (16.2)
	Depressed mood	123 (21.0)
	Missing**	810
Any recorded use of medication, after diagnosis of dementia	Anti-dementia drugs	662 (47.3)
	Antidepressants	422 (30.1)
	Antipsychotics	264 (18.9)
	Sedatives	277 (19.8)
MMSE score at diagnosis	Mean (SD)	18.2 (6.6)
Time between diagnosis and death *– (years)*	Mean (SD)	1.9 (1.7)

KEY: SD = standard deviation; IQR = interquartile range; IMD = Index of Multiple Deprivation; HoNOS = Health of the Nation Outcome Scales; MMSE = Mini-mental state examination

Notes: *HoNOS categories dichotomised to 0–1 (no or minor problem) and 2–4 (problem behaviour)); ** The number of missing data varies according to HoNOS items (between 808 and 810)

### Severity of dementia at death

**Fig. 1:**
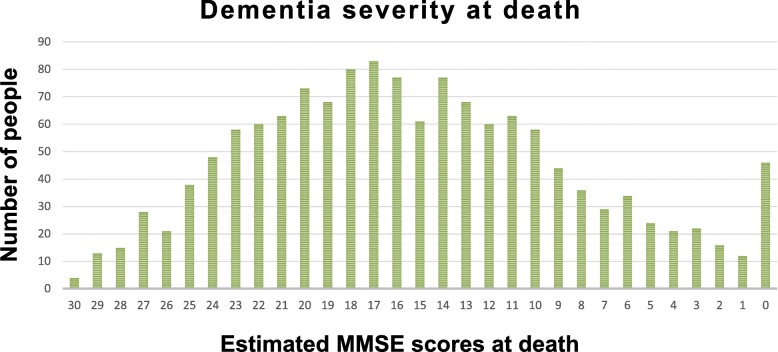
Distribution of mini-mental state examination (MMSE) scores at death (n = 1400)

The mean last MMSE score was 17.0. The mean annual MMSE decline between penultimate and last MMSE assessment was 1.5 points and the mean time from last MMSE score to death was 1.4 years (s.d. = 1.64). The mean estimated MMSE score at death was 15.3 (s.d. = 7.0) (Table 2). We found that 27.4% (95% confidence interval 25.1, 29.8) had severe dementia, 50.4% (47.8, 53.0) had moderate dementia at death, and 22.2% (20.1, 24.5) died with mild dementia (Table 2). The estimated MMSE scores at death were normally distributed except that 46 people had a floor MMSE score estimated as 0 (Fig. 1).

**Table 2:** Distribution of dementia severity at death, using estimated mini-mental state examination scores

		Estimated MMSE scores (n = 1400)	Estimated MMSE scores within 1 year of death (*n* = 782)
*Mean (SD)*		15.3 (7.0)	16.3 (6.8)
Dementia severity n (% (95% CI))	*Mild*	311 (22.2 (20.1, 24.5))	142 (18.2 (15.6, 21.0))
	*Moderate*	706 (50.4 (47.8, 53.0))	389 (49.7 (46.3, 53.2))
	*Severe*	383 (27.4 (25.1, 29.8))	251 (32.1 (28.9, 35.5))

KEY: MMSE = mini-mental state examination

Notes: Mild dementia = estimated MMSE 20–30; moderate dementia = 10–19; Severe dementia = 0–9

In our sensitivity analysis, which included only the 782 people who had their last MMSE within one year of death (Table 2), we found a similar proportion of people had moderate dementia but that a higher proportion (32.1%) had severe dementia at death. The mean MMSE was however, slightly higher (16.3 (s.d. = 6.8)).

### Clinical and demographic predictors of dementia severity at death

In the fully adjusted linear regression model (Table 3), we included 452 people with all the covariates recorded. This showed that women, those of minority ethnicity background, those from more socio-economically deprived backgrounds, and those with agitation died with more severe dementia. Women were estimated to score 1.4 MMSE points lower at death (0.1, 2.8, *p* = 0.04). Similarly, those from a Black, Asian and other ethnic origin scored 3.5 (1.5, 5.5, *p* = 0.001) points, 4.7 (0.5, 9.0, *p* = 0.03) and 5.2 (0.8, 9.7, *p* = 0.02) points lower respectively on the estimated MMSE, compared to White people. Worse score on the IMD was associated with dying with more severe dementia, with each 10 point increase in deprivation score associated with 0.4 points lower on the MMSE (0.0, 0.8, p = 0.04). Having problems with agitation was associated with estimated MMSE 2.5 points (1.0, 4.0, p = 0.001) lower. Conversely, those who were divorced scored 2.2 points (0.0, 4.3, *p* = 0.05) higher on the MMSE. In the regression analysis without the HoNOS variables, which included 1131 people, the results were similar to that of our primary analysis, with the exception that those who were single died with less severe dementia (1.3 points higher on the MMSE (0.1, 2.5, *p* = 0.03) and no association for ‘other’ ethnic origin with dementia severity at death was found. In our sensitivity analysis assessing factors associated with estimated MMSE in the 291 people who had been assessed within one year of death and had complete covariate data (Additional file 1), similar results were obtained, except that ethnicity was no longer associated with dementia severity at death.

**Table 3:** Association between clinical and demographic characteristics and mini-mental state examination scores at death

Characteristic		Univariable analysis			Multivariable analysis (*n* = 452)		
		Coefficient	95% CI	*P*-value	Coefficient	95% CI	P-value
Age at diagnosis (per 1 year increase)		0.0	−0.0 to 0.1	0.20	−0.0	− 0.1 to 0.1	0.75
Sex	Reference category: Male						
	*Female*	−0.8	−1.5 to − 0.1	0.04	− 1.4	−2.8 to − 0.1	0.04
Ethnicity	Reference category: White			0.001*			0.001*
	*Black*	−3.4	−4.8 to − 2.0	0.001	− 3.5	−5.5 to − 1.5	0.001
	*Asian*	− 2.7	− 4.9 to −0.5	0.02	− 4.7	−9.0 to − 0.5	0.03
	*Other*	−0.3	−2.6 to 2.0	0.80	−5.2	−9.7 to − 0.8	0.02
Marital status	Reference category: Married			0.001*			0.26*
	*Widowed*	0.7	−0.3 to 1.7	0.18	0.8	−0.9 to 2.4	0.36
	*Divorced*	1.3	−0.1 to 2.7	0.07	2.2	0.0 to 4.3	0.047
	*Single*	1.8	0.7 to 2.9	0.002	0.7	−1.1 to 2.5	0.45
Dementia type	Reference category: Alzheimer’s disease			0.004*			0.43*
	*Vascular dementia*	−0.2	−1.2 to 0.8	0.71	−1.1	−2.9 to 0.9	0.28
	*Dementia with Lewy bodies*	0.5	−1.9 to 3.0	0.68	1.8	−3.0 to 6.6	0.46
	*Other*	1.8	0.5 to 3.0	0.008	1.0	−1.0 to 3.0	0.33
	*Unspecified*	1.4	0.4 to 2.3	0.004	0.3	−1.2 to 1.9	0.69
IMD (per 10-unit increase in deprivation)		−0.3	−0.5 to −0.0	0.04	− 0.4	−0.8 to − 0.0	0.04
HoNOS at diagnosis – problems with**:	*Agitation*	−2.3	−3.6 to −1.1	0.001	−2.5	−4.0 to −1.0	0.001
	*Hallucinations*	−1.8	−3.3 to −0.4	0.02	−0.7	−2.4 to 1.0	0.43
	*Depressed mood*	−0.3	−1.6 to 1.1	0.70	0.7	−0.8 to 2.3	0.37
Time between diagnosis and death (per 1 year later)		−0.7	−0.9 to − 0.5	0.001	−0.5	−1.1 to 0.0	0.06

KEY: IMD = Index of Multiple deprivation; HoNOS = Health of the Nation Outcome Scales; clinical symptoms were derived from HoNOS scale

Notes: * overall class effect for categorical variables; ** HoNOS categories dichotomised to 0–1 (no or minor problem) and 2–4 (problem behaviour))

#### Association between use of medication and dementia severity at death

Multivariable analysis of the association between different medication use after dementia diagnosis and severity of dementia at death (Table 4), showed that use of antipsychotics was associated with scoring 2.7 points lower on the MMSE at death than individuals who had not taken antipsychotics (0.9, 4.6, *p* = 0.004). Use of other psychotropic medication was not associated with dementia severity at death.

**Table 4:** Association between medication use and mini-mental state examination score at death

Any recorded use of medication after dementia diagnosis	Univariable analysis			Multivariable analysis ^a^ (n = 452)		
	Coefficient	95% CI	P-value	Coefficient	95% CI	P-value
Acetylcholinesterase inhibitors / memantine	−0.6	− 1.3 to 0.2	0.12	− 0.0	− 1.6 to 1.6	0.97
Antidepressants	0.2	−0.7 to 0.9	0.72	0.1	−1.4 to 1.6	0.94
Sedatives	−1.6	−2.5 to −0.7	0.001	−1.1	− 2.8 to 0.7	0.24
Antipsychotics	−2.7	−3.6 to −1.8	< 0.001	− 2.7	−4.6 to −0.9	0.004

Notes: ^a^ adjusted for age, sex, ethnicity, marital status, IMD, dementia type, problems with agitation, hallucinations and depression (from HoNOS scale) and time between diagnosis and death

## Discussion

This is the first study, to our knowledge to report the severity of dementia at death of a cohort of people with dementia. Importantly we find that only one quarter had severe dementia, half had moderate dementia and the rest had mild dementia at death. The mean MMSE at death was 15.3 and we found a normal distribution of estimated MMSE scores at death except for those with estimated MMSE score of 0, a consequence of the floor effect of the MMSE assessment.

This result was unexpected as we had anticipated that most people in the cohort would die with severe dementia. It suggests that most of our cohort died from other illnesses rather than solely from the consequences of advanced dementia, although the presence of dementia may have led to a worse prognosis than others with the same illness [21]. The age-specific incidence of dementia has decreased in the UK over the past 20 years [22], meaning that the condition occurs later now than in previous generations, and is therefore more likely to coexist with other illnesses. Around 70% of people with dementia have at least two comorbid chronic diseases and these increase the risk of hospitalisation and mortality [23]. This elevated mortality may have contributed to a large proportion of our cohort dying with milder forms of the disease. Furthermore, a major drive in dementia policy in the UK and other countries has been early diagnosis of dementia [24] and the services whose data we used have been successful at increasing dementia diagnosis [25], so our naturalistic cohort includes many who had dementia diagnosed at very mild stages, who may have died from other conditions, with less severe dementia.

The mean MMSE was slightly lower in the whole sample than in our sensitivity sub-group of people assessed within one year of their death, as the whole cohort included a higher number of people (46 v 24 people) whose estimated MMSE was 0, due to a long interval between their last MMSE and death. In the sensitivity analysis, a higher proportion of people had severe dementia than in the main analysis, which may be because our method underestimated the decline, which may be more precipitous before death, or because people with more advanced dementia were more likely to have been seen and cognitively assessed by the clinical service close to death.

More severe dementia at death was associated with being female, from ethnic minority background, more socioeconomically deprived, showing symptoms of agitation and taking antipsychotic medication. Divorced people died with less severe dementia. Our observational analyses of the association between sociodemographic and clinical factors and dementia severity cannot establish a causal relationship. The identified factors may cause dementia to progress more rapidly; they may indicate people with lower cognitive reserve [26] and therefore more severe cognitive impairment at the same pathological stage of the disease; or they may indicate those with better physical health who therefore live longer with dementia and have the condition more severely when they die.

We found that women died with more severe dementia than men, which may result from this cohort of women having received less education [27] and therefore having less cognitive reserve so showing more severe cognitive symptoms for the same level of pathology, or be a consequence of their greater life-expectancy, allowing more dementia progression before death. Our finding that people from minority ethnic background died with more severe dementia than their white counterparts may also reflect lower cognitive reserve related to less education [28]. In addition, people from ethnic minority backgrounds develop dementia at a younger age [29] and may therefore live longer with the disease. A final explanation is that people from minority groups present later [30], so those who die with less severe illness may never have presented to memory services. Alternatively, people from ethnic minority origins may engage less with management of their condition [31], thus experience a more precipitous cognitive decline. Higher neighbourhood level socioeconomic deprivation being associated with more severe dementia at death is also likely to result from lower cognitive reserve and younger development of dementia [32]. Our finding that divorced individuals died with less severe dementia compared to married people may be due to the mortality risks associated with being divorced [33] meaning divorced individuals died earlier and with milder dementia than married people.

Our results showed agitation to be associated with more severe dementia at death and this is likely to be because agitation at diagnosis is a consequence of more severe dementia, which increases in severity as dementia progresses [34]. An alternative is that agitation caused more rapid dementia progression, supported by studies suggesting that neuropsychiatric symptoms increase neuropathological burden [35]. Antipsychotics are used for psychosis or sometimes agitation, which are markers of more severe dementia, although the potential contribution of antipsychotic use to cognitive decline [36] needs further exploration.

### Strengths and limitations

This is the first study, to our knowledge, to evaluate dementia severity at death and examine demographic and clinical predictors. Our database allowed analysis of all routinely collected clinical data, with no requirement for explicit consent to involvement in the database, meaning that we had a large naturalistic cohort of people who had been clinically-diagnosed with dementia before dying. Our results are likely to be representative of people with clinically-diagnosed dementia living in similar areas, as memory services are the mainstay of UK dementia diagnosis and assessment [24] and CIFT has a high estimated diagnosis rate – 84% of people in the catchment area estimated to have dementia from epidemiological studies, have a formal diagnosis [25] – but our findings may not generalise to people with dementia who have not been diagnosed, who may have been more likely to die with milder dementia.

Our study has limitations; the use of electronic health records which were not collected for research purposes meant that we could only adjust for routinely recorded factors. We would have liked to adjust for education, which would be a more sensitive marker of cognitive reserve than socioeconomic deprivation, and would have ideally had more sensitive measures of physical-ill health, enabling us to examine in detail the potential confounding effect of physical illness on dementia severity at death. Our analysis assumed a linear decline in MMSE at end of life, and applied the average MMSE decline to the whole sample, whereas this may not be the case. Rate of decline likely depends on a number of factors including initial severity, type of dementia, age, neuropsychiatric symptoms, medication, sex and education and we did not have data on all these domains to allow us to predict decline for each patient individually, so we used the mean rate of decline in the sample. The mean annual rate of decline in our sample was 1.5 MMSE points. One study reported 6 month MMSE decline of 0.9 points [37], whilst others have reported an annual decline of 2.2–2.3 points [38, 39], although these studies only examined people with Alzheimer’s disease, whereas we included people with all dementia types and the rate of decline differs according to dementia type [40]. Finally, for some patients, it is possible that MMSE testing was abandoned when they had more severe dementia as the patient may struggle to complete testing and/or the clinician may deem that it does not add significant information, although clinicians would often record the MMSE score at the point of abandoning the test; however, reduced recording of MMSE in severe dementia may mean that dementia severity is underestimated in this study.

## Conclusions

We found the majority of people in the sample died with moderate dementia and only one quarter had severe dementia at death. This study provides important information for clinicians and the public about the prognosis of people with dementia and should inform the development of end-of-life services tailored to the condition. Dying with severe dementia is a major concern for people with early disease [41], but we found that this occurs less frequently than expected. People with dementia, their family carers, and healthcare professionals involved in their care may delay decisions related to end-of-life, assuming this may not be relevant, and research suggests that take-up of advance care plans has been low; [42] however, our research supports the need for professionals to encourage early discussions about who may help with decisions and end-of-life preferences. All palliative care in older people should consider if the individual has dementia and how this affects their decision-making ability, the possible treatments and their ability to carry out a plan without help. People with mild to moderate dementia often appear unimpaired in social contexts to people who do not know them and it is easy to miss the disability that their cognitive impairment may cause. Clinicians involved in end of life care should consider the presence of dementia in people with less obvious signs of the condition and take this into account when explaining clinical information and making treatment decisions.

## Additional file

Additional file 1: **Appendix 1.** Association between clinical and demographic characteristics and the mini-mental state examination at death, using only scores recorded within one year of death. **Appendix 2.** Flow diagram of study patient inclusion/exclusion. (DOCX 41 kb)

## Abbreviations

CIFT: Camden and Islington NHS Foundation Trust
CRIS: Clinical Record Interactive Search
GATE: General Architecture for Text Engineering
HoNOS: Health of The Nation Outcome Scales
ICD: International Classification of Diseases
IMD: Index of multiple deprivation
LSOA: Lower super output area
MCI: Mild Cognitive Impairment
MMSE: Mini-Mental State Examination
NLP: Natural language processing
PPR: Post processing rule
PPV: Positive predictive value
