# Supplementary material for: Dementia severity at death: a register-based cohort study
Source: BMC Psychiatry. 2018 Nov 1;18:355. doi: 10.1186/s12888-018-1930-5 (PMC6211473; doi:10.1186/s12888-018-1930-5)
Supplement: Supplementary file 1 — Appendix 1. Association between clinical and demographic characteristics and the mini-mental state examination at death, using only scores recorded within one year of death. Appendix 2. Flow diagram of study patient inclusion/exclusion. (DOCX 41 kb) [file 12888_2018_1930_MOESM1_ESM.docx]

# Appendices

**Appendix 1: Association between clinical and demographic characteristics and the mini-mental state examination at death, using only scores recorded within one year of death**

| **Characteristic** |  | **Univariable analysis** | | | **Mutually adjusted multivariable analysis (n=452)** | | |
| --- | --- | --- | --- | --- | --- | --- | --- |
|  |  | **Coefficient** | **95% CI** | **P-value** | **Coefficient** | **95% CI** | **P-value** |
| **Age at diagnosis (per 1 year increase)** | | 0.0 | -0.4 to 0.1 | 0.386 | 0.03 | 0.1 to 0.2 | 0.631 |
| **Sex** | **Reference category: Male** |  |  |  |  |  |  |
|  | *Female* | -0.5 | -1.5 to 0.5 | -0.5 | -2.3 | -4.0 to -0.6 | 0.009 |
| **Ethnicity** | **Reference category: White** |  |  | *0.009** |  |  | *0.172** |
|  | *Black* | -3.0 | -4.8 to -1.1 | 0.002 | -1.2 | -3.8 to 1.3 | 0.330 |
|  | *Asian* | -2.1 | -5.1 to 0.9 | 0.162 | -3.6 | -9.0 to 1.8 | 0.187 |
|  | *Other* | -1.0 | -4.1 to 2.1 | 0.525 | -4.8 | -10.7 to 1.1 | 0.108 |
| **Marital status** | **Reference category: Married** |  |  | *0.076** |  |  | *0.111** |
|  | *Windowed* | 0.7 | -0.6 to 2.0 | 0.287 | 1.8 | -0.3 to 3.8 | 0.088 |
|  | *Divorced* | 1.8 | -0.1 to 3.7 | 0.061 | 3.1 | 0.4 to 5.7 | 0.023 |
|  | *Single* | 1.9 | 0.3 to 3.5 | 0.022 | 1.5 | -0.8 to 3.7 | 0.192 |
| **Dementia type** | **Reference category: Alzheimer’s disease** |  |  | *0.414** |  |  | *0.385** |
|  | *Vascular dementia* | -0.5 | -1.8 to 0.9 | 0.522 | -1.8 | -4.5 to 0.8 | 0.178 |
|  | *Dementia with Lewy bodies* | 0.3 | -2.7 to 3.3 | 0.848 | 1.0 | -3.9 to 5.8 | 0.693 |
|  | *Other* | 1.3 | -0.4 to 3.0 | 0.137 | 1.1 | -1.4 to 3.7 | 0.379 |
|  | *Unspecified* | 0.6 | -0.6 to 1.7 | 0.346 | 0.4 | -2.2 to 3.0 | 0.656 |
| **IMD (per 10-unit increase in deprivation)** | | -0.4 | -0.7 to -0.1 | 0.012 | -0.6 | -1.1 to 0.1 | 0.015 |
| **Clinical symptoms: presence of** | *Agitation* | -2.4 | -4.0 to -0.9 | 0.002 | -2.1 | -4.0 to -0.1 | 0.029 |
|  | *Hallucinations* | -2.7 | -4.4 to -1.0 | 0.002 | -1.7 | -3.9 to 0.4 | 0.109 |
|  | *Depressed mood* | -0.8 | -2.4 to 0.9 | 0.352 | 1.2 | -0.8 to 3.1 | 0.248 |
| **Time between diagnosis and death (per 1 year later)** | | -0.4 | -0.8 to -0.1 | 0.020 | -0.2 | -0.9 to 0.6 | 0.690 |

KEY: IMD= Index of Multiple deprivation; HoNOS = Health of the Nation Outcome Scales; clinical symptoms were derived from HoNOS scale

Notes: * overall class effect for categorical variable

# **Appendix 2: Flow diagram of study patient inclusion/exclusion.**

Dementia records extracted though unstructured (GATE) fields in CRIS

(n= 400)

Dementia records extracted though structured fields in CRIS

(n=1409)

Individuals whose final diagnosis was not dementia, i.e. MCI (n=15)

d RECORDS REMOVED

Total dementia records extracted (n=1809)

Individuals without MMSE score (i.e. only ID number and dementia diagnosis) (n=7)

Individuals with dementia diagnosis before age 65 (n=4)

Individuals with MMSE score outside the potential range of scores, (i.e. 0 to 30) (n=3)

Individuals who had their MMSE score recorded more than a year after death (n=70)

Final cohort – Records which met all the inclusion criteria – (n=1400)

Individuals who had multiple MMSE scores recorded on the same day (n=310)
